# Severe form of an acute schistosomiasis with involvement of the gastrointestinal tract and lungs complicated by Clostridioides difficile infection

**DOI:** 10.1007/s11739-025-03929-z

**Published:** 2025-05-06

**Authors:** Piotr Zieliński, Małgorzata Sulima, Robert Pleśniak, Katarzyna Sikorska, Beata Szostakowska

**Affiliations:** 1 Division of Tropical & Parasitic Diseases, Medical University of Gdańsk, Gdańsk, Poland; 2 Division of Tropical Medicine & Epidemiology, Medical University of Gdańsk, Gdańsk, Poland; 3 Clinical Department of Infectious Diseases, College of Medical Sciences, Medical Centre in Łańcut, University of Rzeszów, Rzeszow, Poland; 4 Division of Tropical Parasitology, Medical University of Gdańsk, Gdańsk, Poland

A 32-year-old woman, who returned from a 6-week trip to Guinea, was admitted to the hospital due to fever, abdominal pain, persistent watery diarrhoea and cachexia with a BMI of 14. During her stay in Guinea, she ate local food and drank water, and swam in freshwater bodies. Before leaving, she was vaccinated against hepatitis A, typhoid fever and yellow fever. She took doxycycline for 5 weeks as anti-malarial prophylaxis. Initially, after admission to the first hospital in Łańcut due to suspected traveler’s diarrhea, azithromycin and metronidazole were administered orally for 2 days. After a 3-day hospitalization in Łańcut, due to lack of clinical improvement and suspicion of a tropical disease, the woman was transported to the hospital in Gdynia. Upon admission to the hospital in Gdynia, laboratory tests revealed high white blood cells count (WBC: 12.38 G/L), increased neutrocytosis (8.4 G/L), lymphopenia (0.91 G/L), eosinophilia of 1.33 G/L (N 0.04–0.40 G/L), anaemia (HGB 11.3 g/dl), high CRP level of 109 mg/L (N < 5 mg/L), high procalcitonin level of 1.24 ng/ml, hypocalcemia (7.4 mg/dl), hypoalbuminemia (2.8 g/l), increased activity of transaminases ALAT 150 IU/L (N < 34 IU/L), ASPAT 114 IU/L (N < 31 IU/L) and GGTP 216 IU/L (N 8–33 IU/L). Stool tests confirmed the presence of toxins and the *C. difficile* GDH antigen. Stool examination performed using the RT-PCR method for the most common intestinal pathogens was negative. The treatment included vancomycin 4 × 500 mg orally and metronidazole 3 × 500 mg intravenously, initially in combination with ceftriaxone, which was then discontinued after the second day when negative microbiological results of blood and urine tests were obtained. In addition, parenteral nutrition, intravenous fluid therapy, antipyretic drugs, proton pomp inhibitors, and fluconazole were administered. The patient’s general condition improved to some extent. On the 4th day of hospitalisation, the diarrhoea subsided; however, high fever of up to 39 degrees Celsius and abdominal pain persisted. CT scan of the abdominal cavity revealed hepatosplenomegaly, thickening of the walls of the rectum, sigmoid colon and descending colon with increased density of fatty tissue around the walls and widening of the lumen of the transverse and ascending colon up to 4.5 cm. In addition, numerous scattered intralobular, ill-defined nodules measuring 1–4 mm in size were visible bilaterally in the lungs in the area covered by diagnostic imaging. A CT scan of the chest revealed new lesions located mainly subpleurally in the dorsal segments, presenting as areas of confluent consolidation, among which there were clusters of small nodules. Some nodules were surrounded by peri-nodular halo and ground-glass opacities (Fig. 1) [1, 2]. The patient did not present any respiratory symptoms. The material obtained from bronchoalveolar lavage was tested negative for tuberculosis (direct preparation, PCR and cultures). A positive serological blood test result for schistosomiasis was obtained in the ELISA test (NovaTec) and the result was confirmed in the Western Blot test (LDBIO Diagnostics). Parasitological tests of stool and urine were negative. The treatment included praziquantel 40 mg/kg body weight together with artemether 6 mg/kg b.w. divided in 3 doses every 12 h and prednisone 40 mg/d for 5 days [3, 4]. Due to severe illness, colonoscopy was not performed at the moment of diagnosis of CDI [5, 6]. For clinical evaluation and therapeutic decisions authors used clinical guidelines for CDI of Polish Society of Epdiemiology and Infectious Diseases [7]. Vancomycin 500 mg qid orally and Metronidazol intravenously are recommended in the fulminant course of CDI according to these recommendations. In the presented case laboratory criteria for fulminant course of CDI (according to polish guidelines and European Society of Clinical Microbiology and Infectious Diseases guidelines) were not fulfilled [6, 7, 8]. However, based on the clinical picture and severe course of the disease with recurrence of high fever, rapid clinical deterioration, fearing of rapid progression of CDI, before the results of the tests for schistosomiasis were obtained, we decided to use the protocol of treatment with high doses of vancomycin with intravenous metronidazol. We concluded that the patient was also in high-risk group for the recurrence of C. difficile infection, similar to the patients with inflammatory bowel disease [9]. Vancomycin prolonged treatment is not recommended in the first episode of C. difficile infection, therefore the treatment was applied after obtaining patient’s consent for this treatment regimen. As a part of the treatment, the patient received steroids and the presence of inflammatory lesions in the colon in the course of schistosomiasis was suspected, which was confirmed later in endoscopy. Colonoscopy revealed granular lesions in the wall of the large intestine, and PCR testing confirmed the presence of S. mansoni genetic material. Oral vancomycin treatment was continued 500 mg 4 times a day for 14 days, then 125 mg once a day for 7 days and 125 mg every other day for last 7 days duration therapy. The fever subsided on the next day after the administration of anti-parasitic drugs, and the abdominal pain also disappeared. The patient’s clinical condition improved. In the follow-up blood count, an increase in eosinophilia was observed to 2.43 G/L (N 0.04–0.40 G/L). The patient was discharged home. One month later, she received a second dose of praziquantel. In a follow-up chest CT scan, significant regression of lung lesions was observed (Fig. 1). The suspicion of schistosomiasis was based on epidemiological data. The patient visited Guinea (Africa), an endemic area for schistosomiasis. WHO has implemented targeted treatment with praziquantel through the large-scale treatment (preventive chemotherapy) of affected populations in Guinea [10]. This is an interesting case of a patient with gastrointestinal symptoms and *C. difficile* infection, who was diagnosed with acute pulmonary schistosomiasisThis is an interesting case of a patient with gastrointestinal symptoms and *C. difficile* infection, who was diagnosed simultaneously with acute schistosomiasis and involvement of lungs and gastrointestinal tract. Informed consent for the publication of the case report was obtained from the patient.

**Fig. 1:**
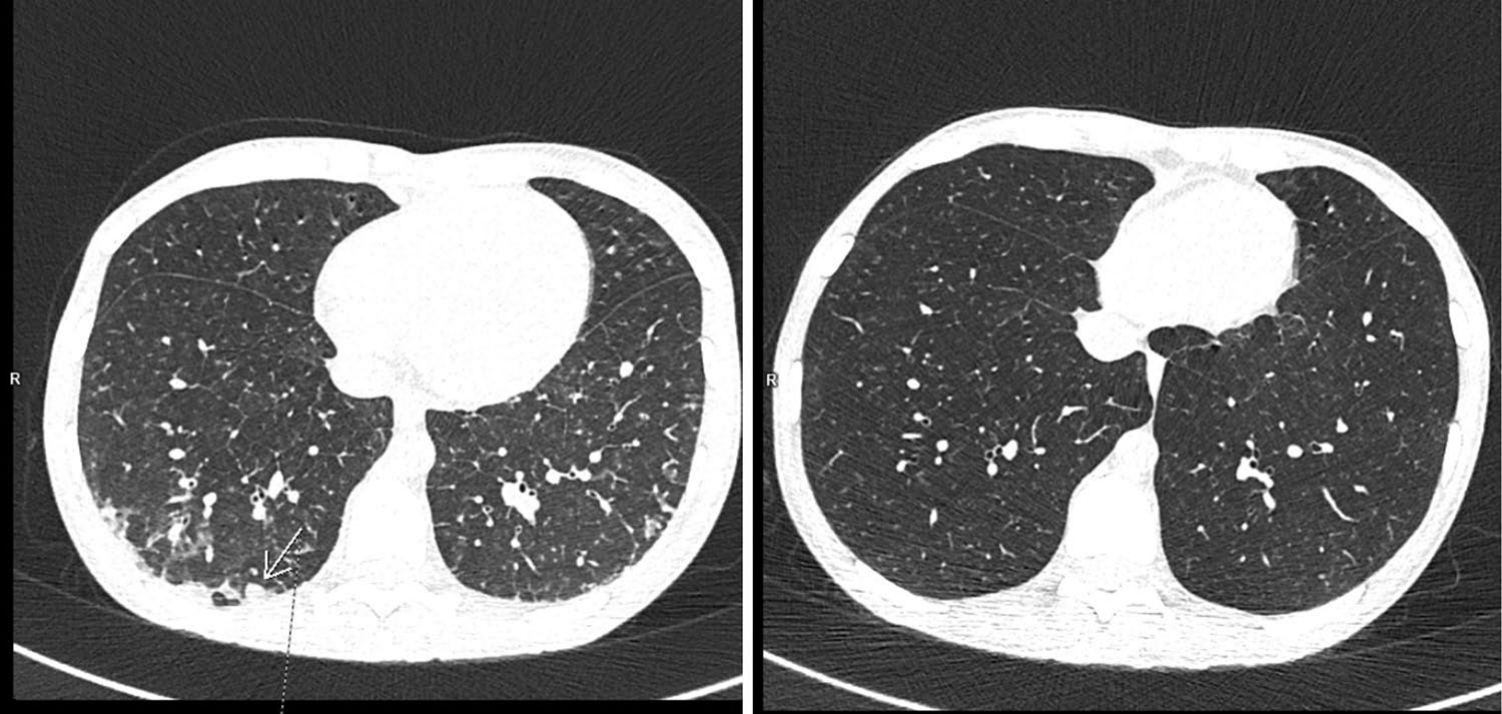
Left side: First CT scan. Numerous intralobular small nodules disseminated bilaterally, white arrow—one of them located near the thoracic wall. Right side: Second CT scan, the same area after treatment

## Data Availability

Data are not included. Data available due to sensitive nature with special permission at Division of Tropical & Parasitic Diseases, Medical University of Gdańsk and Clinical Department of Infectious Diseases, College of Medical Science, University of Rzeszów.
